# Changes in patterns of uveitis at a tertiary referral center in Northern Italy: analysis of 990 consecutive cases

**DOI:** 10.1007/s10792-016-0434-x

**Published:** 2017-01-09

**Authors:** Cimino Luca, Aldigeri Raffaella, Marchi Sylvia, Mastrofilippo Valentina, Viscogliosi Fabiana, Coassin Marco, Soldani Annamaria, Savoldi Luisa, De Fanti Alessandro, Belloni Lucia, Zerbini Alessandro, Parmeggiani Maria, Chersich Matthew, Soriano Alessandra, Salvarani Carlo, Fontana Luigi

**Affiliations:** 1 Immunology Eye Unit, Eye Department, Arcispedale S. M. Nuova-IRCCS, Reggio Emilia, Italy; 2 Department of Clinical and Experimental Medicine, University of Parma, Parma, Italy; 3 Eye Department, Arcispedale S. M. Nuova-IRCCS, Reggio Emilia, Italy; 4 Scientific Directorate, Arcispedale S.M. Nuova-IRCCS, Reggio Emilia, Italy; 5 Department of Pediatrics, Arcispedale S. M. Nuova.IRCCS, Reggio Emilia, Italy; 6 Clinical Immunology, Allergy and Advanced Biotechnologies Unit, Arcispedale Santa Maria Nuova-IRCCS, Reggio Emilia, Italy; 7 Wits Reproductive Health and HIV Institute, Faculty of Health Sciences, University of the Witwatersrand, Johannesburg, South Africa; 8 Rheumatology Unit, Department of Internal Medicine, Arcispedale S. M. Nuova-IRCCS, Reggio Emilia, Italy

**Keywords:** Italy, Epidemiology, Infection, Interdisciplinary approach, Systemic disease, Uveitis

## Abstract

**Purpose:**

The role of uveitis, an uncommon ocular disease, is often neglected in research and treatment of autoimmune conditions. The study described the spectrum of uveitis at a referral center in North Italy, and compared that to a previously published series of patients.

**Methods:**

We reviewed all patients with uveitis diagnosed from 2013 to 2015 at the Immunology Eye Unit, Arcispedale S. M. Nuova-IRCCS, Reggio Emilia, Italy. We examined patient characteristics, disease spectrum, and etiologies.

**Results:**

In total, 990 cases of uveitis were identified, who were mostly female (59%) with a median age at presentation of 44 years (interquartile range = 29–57). Anterior uveitis was most frequent (53.5%), followed by panuveitis (22.8%), posterior (16.2%), and intermediate uveitis (5.5%). Anterior herpetic uveitis (15.6%), Fuchs uveitis (9.7%), and HLA-B27 positive anterior uveitis (7.7%) were the most common specific diagnoses. Compared with the previous series, we observed an increased incidence of uveitis, and a different pattern of diagnoses. Rates of herpetic, HLA-B27 positive uveitis, and presumed ocular tuberculosis were higher, but Fuchs uveitis was less frequent.

**Conclusions:**

The pattern of uveitis appears to be changing, very likely due to population-level increases in infectious diseases, to the availability of new diagnostic tests and to the interdisciplinary approach used in patient diagnosis.

## Introduction

Uveitis represents a heterogeneous group of intraocular diseases, encompassing a range of inflammatory eye conditions affecting not only the uvea, but also the retina, the optic nerve, and the vitreous. Although a rare disease, its prevalence is rising [1–3]. Uveitis can vary substantially in terms of its clinical course, management, and prognosis. While certain types of uveitis require no treatment, have a self-limited course, and a favorable prognosis, other forms carry a considerable risk of vision loss. Uveitis can be limited to the eye or associated to systemic diseases. Conditions can be idiopathic, autoimmune, or caused by many different infectious agents. Since uveitis cases are often not clinically recognized, the diagnostic delay may increase the risk of irreversible sight-threatening complications. Also, as genetic, ethnic, geographic, socioeconomic, nutritional, and environmental factors influence the pathogenesis of uveitis, it is important to assess the patterns of the condition in different geographic regions over time [3, 4]. Drawing on a computerized register of all cases of uveitis at a tertiary-level referral center in Northern Italy, we analyzed the spectrum of uveitis patients from November 2013 to October 2015 and compared it to our previous published series [5].

## Materials and methods

In November 2013, a database was developed to systematically collect data on uveitis patients referred to our Immunology Eye Unit in Reggio Emilia, Italy. We here review the database entries of patients with uveitis referred to our unit over 24 months. Human Immunodeficiency Virus (HIV) positive and masquerade uveitis patients were excluded. All patients gave written informed consent for analysis of their data, and the study was approved by the Arcispedale S. Maria Nuova-IRCCS Institutional Review Board.

Demographic data, medical history, ocular symptoms and signs, special investigations and therapy of patients were analyzed. At presentation, all patients underwent the following: best-corrected Snellen visual acuity, applanation tonometry, slit-lamp evaluation of the anterior segment, and fundus examination. When appropriate, fluorescein angiography, indocyanine green angiography, optical coherence tomography, ocular ultrasonography, and visual field testing were performed. Furthermore, when required, patients underwent complete diagnostic work-up, including laboratory tests and medical evaluation. The selection of diagnostic work-up was based on the positive predictive value of the laboratory tests. For example, as HLA-B27-positive anterior uveitis does not have a granulomatous component, in patients who are HLA-B27 positive and have anterior granulomatous uveitis, two distinct pathologies were considered (for example, herpetic uveitis with concurrent HLA-B27 positivity).

The anatomical classification and final diagnosis were based on the criteria of the International Uveitis Study Group (IUSG) and the Standardization of Uveitis Nomenclature (SUN) working group [6, 7]. Furthermore, granulomatous and non-granulomatous uveitis were categorized from a clinical (and not histological) point of view, a useful distinction in clinical practice, as some uveitis conditions are always non-granulomatous, such as HLA-B27 or Behçet-related uveitis. The clinical aspects of non-granulomatous uveitis are the presence of fine endothelial keratic precipitates (dots), in the absence of iris nodules and/or choroidal granulomas, and granulomatous if larger (“mutton fat”) keratic precipitates, Koeppe and/or Busacca nodules and/or choroidal granulomas [8]. Patients were referred to relevant specialists if systemic disease was suspected. Criteria for specific diagnosis included a defined etiology, typical clinical appearance, and history or classification based on pathological or diagnostic laboratory parameters.

Here, we briefly illustrate our approach for making specific diagnoses and, when necessary, interdisciplinary work-up.

### HLA-B27-positive anterior uveitis

Patients with acute anterior uveitis, always clinically non-granulomatous, and typed for the presence of the HLA-B27, regardless of the presence of systemic disease.

### Anterior herpetic uveitis

Intraocular inflammation that is usually acute, unilateral, and granulomatous with posterior synechiae and sectoral iris atrophy [5]. In selected cases, where clinical presentation is atypical and/or recurrent despite antiviral therapy, we performed both the serological profile of the suspected microorganism, Herpes Virus-1 (HSV-1), Herpes Virus-2 (HSV-2), Varicella Zoster Virus (VZV), and Real-Time Polymerase Chain Reaction (RT-PCR) on the aqueous tap [9, 10]. In case of clinical suspicion, but negative result of RT-PCR, antibody index coefficient (AIC) (Improved Goldmann–Witmer index) assessment is performed [9, 11, 12].

### Anterior cytomegalovirus (CMV) uveitis

In the case of granulomatous anterior uveitis, unilateral, recurrent episodic uveitis, without sectorial iris atrophy, with raised intraocular pressure, open angle, immunocompetent patients, after the assessment of seropositivity for CMV infection, we confirm the diagnosis with an aqueous tap for RT-PCR analysis, and Antibody Index testing in case of negative results of RT-PCR [11–13].

### Fuchs uveitis

It is mostly unilateral granulomatous uveitis involving the anterior segment and the vitreous body. The main clinical signs are characteristic sparsely distributed stellate granulomatous keratic precipitates, iris stroma atrophic changes, the absence of synechiae, anterior vitritis, and the absence of cystoid macular oedema [5].

### Toxoplasmosis “typical retinochoroiditis”

The diagnosis is based on a compatible fundus lesion and positive serology for toxoplasma antibodies. A solitary inflammatory focus near an old pigmented scar is typical [5]. If the diagnosis is uncertain in patients seropositive for toxoplasmosis, an aqueous sample is used to confirm the diagnosis with RT-PCR [7, 12].

### Acute retinal necrosis (ARN)

The diagnosis was based on the criteria of American Uveitis Society [8].

### Sarcoidosis

The presumed diagnosis is made on consistent clinical findings with two of the following three diagnostic criteria: elevated angiotensin converting enzyme or lysozyme, cutaneous anergy, and/or with positive chest tomography [14]. We prefer to obtain diagnostic confirmation directly by a positive mediastinoscopic lymphonodular biopsy rather than by interlocutory tests (such as bronchoalveolar lavage) or trans-bronchiolar biopsy (sometimes difficult to obtain).

### Pars planitis

It commonly indicates a subset of intermediate uveitis where there is snowbank or snowball formation occurring in the absence of an associated infection or systemic disease (that is, “idiopathic”) [7].

### Behçet’s disease (BD)

We used the International Study Group (ISG) criteria for BD [15], which includes the presence of recurrent oral ulcers plus two of the following: (a) recurrent genital ulcers, (b) eye lesions, (c) skin lesions, and (d) positive pathergy test.

### Presumed tuberculous (TB) uveitis

We define any kind of uveitis compatible with a tuberculous etiology with Quantiferon Gold TB positivity [16] or Mantoux tuberculin skin test (2U) >15 mm diameter of induration at 48–72 h, with or without abnormalities on chest X-ray, exclusion of other possible causes of uveitis, and the response to anti-tuberculosis treatment [5].

### Primary Inflammatory Choriocapillaropathies (PICCP)

The common denominator in PICCP is choriocapillaris non-perfusion and secondary ischaemia of the outer retina. Autofluorescence (FAF), Fluoroangiography (FA), and Indocyanine green angiography (ICG) are fundamental diagnostic tests for detecting choriocapillaris non-perfusion [17]. The clinical differences between the PICCP could possibly be explained by the level and the severity of the inflammation of the choriocapillaris circulation. Before the diagnosis of PICCP is made, an infectious cause, a neoplastic process, or a systemic vasculitis must be ruled out. The classification of PICCP into any of the known and well-defined entities is useful in predicting evolution and defining therapy [5, 18]: multiple evanescent white dot syndrome (MEWDS), acute posterior multifocal placoid pigment epitheliopathy (APMPPE), multifocal choroiditis, and serpiginous choroiditis.

### Vogt–Koyanagi–Harada (VKH) disease

It is a multisystem disease. Chronic, bilateral, granulomatous panuveitis are associated with the central nervous system, auditory and integumentary manifestations. Its hallmark is bilateral multifocal exudative retinal detachments, hyperemia, and edema of the optic disk. We include lumbar puncture when necessary at the onset of the disease, especially in patients without systemic steroid therapy showing cerebrospinal fluid (CSF) pleocytosis [19, 20].

The term idiopathic is used whenever the intraocular inflammation cannot be attributed to a specific diagnosis.

Descriptive data are presented as mean and standard deviation (SD), or median and interquartile range (IQR), where appropriate. Categorical variables are expressed as frequency and percentages. To assess differences among groups, Student’s *t* test and ANOVA were used for continuous data, and Chi–square tests were conducted for categorical variables. Statistical analysis was performed using SPSS (IBM SPSS statistics v.22).

## Results

Nine-hundred and ninety consecutive patients with uveitis (1481 eyes) diagnosed from November 1, 2013 to October 31, 2015 were included. The majority were female (*n* = 587, 59%; Table 1). There were 120 (12%) cases among children (≤18 years), while 713 (72%) predominated in those aged 19–64, and 157 (16%) in those aged above 65 (*p* < 0.001). Age at onset of uveitis was a median 39 (IQR 24-54), while age at presentation at our clinic was a median 44 years (IQR 29–57). The majority was of Italian ethnicity (884, 89%). Of these, 57% were resident in Emilia Romagna region, 79% in other parts of Northern Italy, and 21% in Central–Southern Italy.

**Table 1:** Demographic and clinical characteristics of patients, by time period and anatomical site

Variable	Cases 2002–2008 (*N* = 1064)	Cases 2013–2015					*P*
		Total cases *N* = 990	Anterior *N* = 530	Intermediate *N* = 74	Panuveitis *N* = 226	Posterior *N* = 160	
Median age at presentation years (IQR)	40 (26–55)	44 (29–57)*	45 (32–59)	26 (15–46)	45 (30–58)	42 (29–55)	<0.001
Gender							0.300
Male	481 (45)	403 (41)*	204 (38)	36 (49)	93 (41)	70 (44)	
Female	583 (55)	587 (59)	326 (62)	38 (51)	133 (59)	90 (56)	
Site							<0.001
Unilateral	825 (78)	499 (50)*	364 (69)	13 (18)	57 (25)	65 (41)	
Bilateral	239 (22)	491 (50)	166 (31)	61 (82)	169 (75)	95 (59)	
Granulomatous							<0.001
Yes	553 (52)	545 (55)	312 (59)	13 (18)	147 (65)	73 (46)	
No	511 (48)	445 (45)	218 (41)	61 (82)	79 (35)	87 (54)	
Non-Italian ethnicity	31 (3)	106 (11)*	43 (8)	6 (8)	44 (19)	13 (8)	<0.001
Has a specific diagnosis	787 (74)	759 (77)	404 (76)	45 (61)	177 (78)	133 (84)	0.002
Infectious etiology	257 (24)	301 (30)*	173 (43)	3 (7)	68 (38)	57 (43)	<0.001
Systemic disease	na	259 (26)	114 (21)	12 (16)	110 (49)	23 (14)	<0.001

* Indicates *P* value <0.05 for comparison between previous cases and current. Data are reported as median (interquartile range) or *n* (%)

Anterior uveitis was the most frequent (53.5% of cases), followed by panuveitis (22.8%), posterior (16.2%), and intermediate uveitis (7.5%). There were considerable differences in disease location of uveitis in terms of age, but not of gender (Table 1). Unilateral involvement was significantly higher in anterior uveitis (69%) than in other anatomical sites. Granulomatous forms were especially prevalent in anterior and panuveitis (59 and 65%, respectively). Panuveitis was the more frequent among patients of non-Italian ethnicity.

**Fig. 1:**
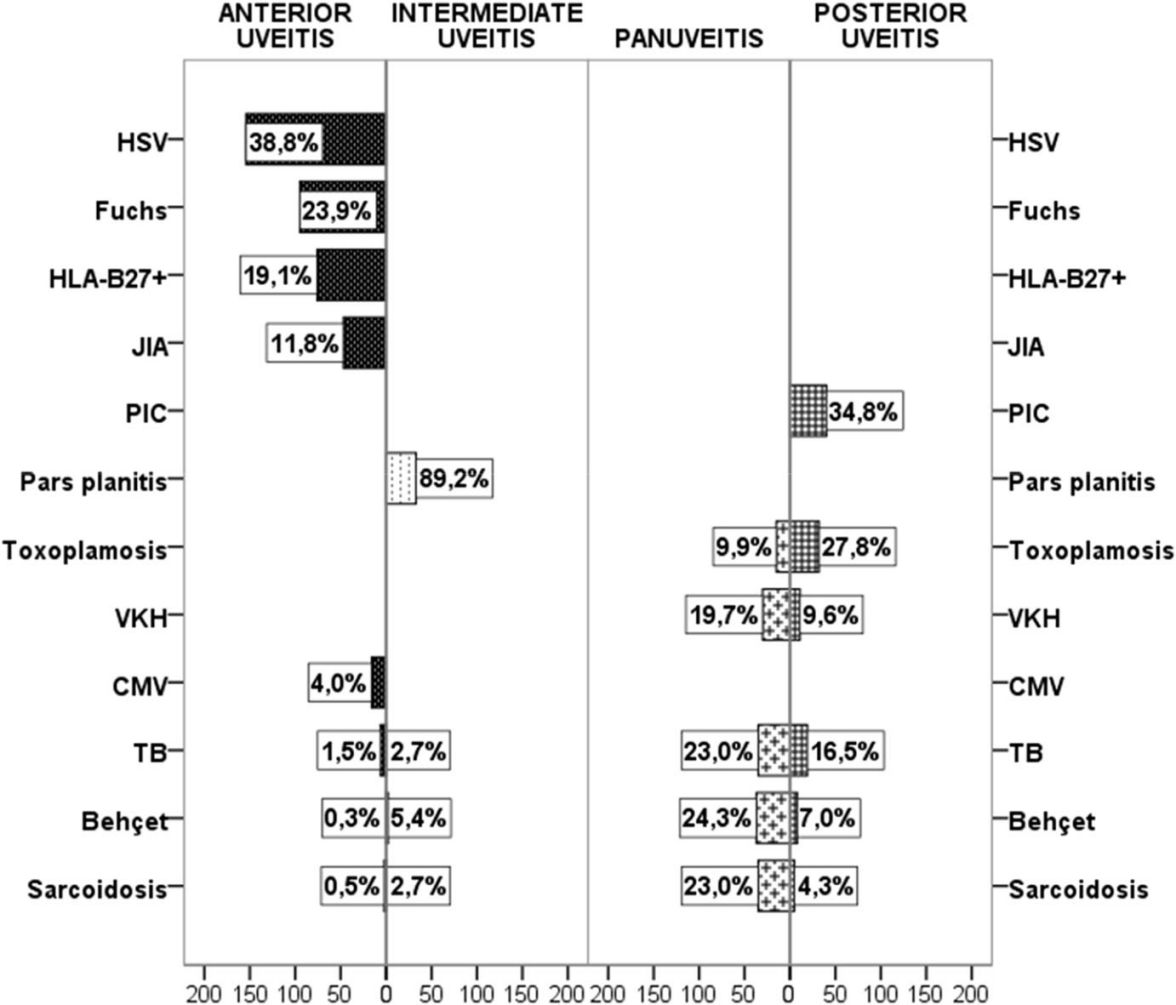
Distribution of the most frequent specific diagnoses according to anatomical classification

**Fig. 2:**
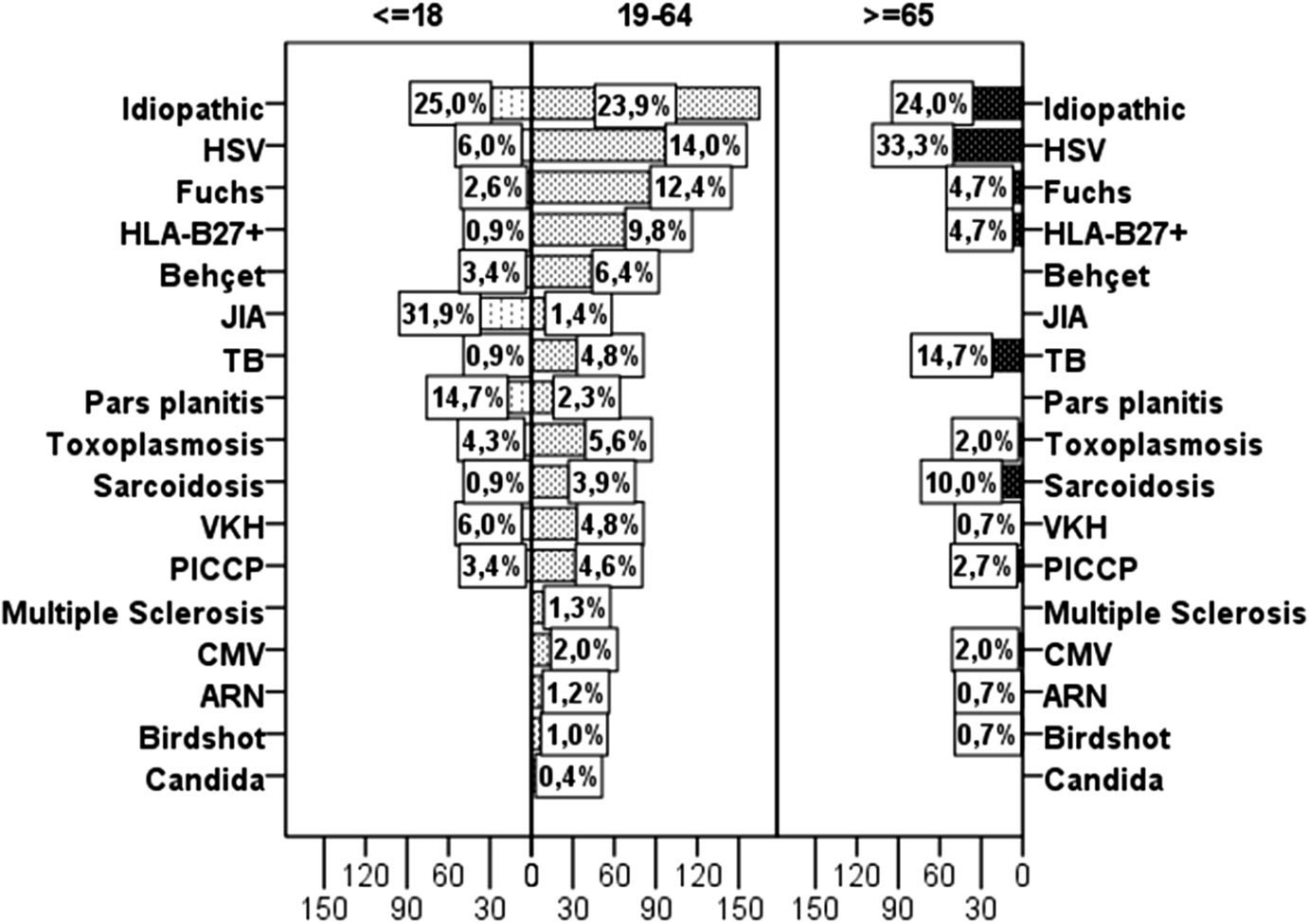
Age distribution of most frequent diagnoses

An etiological diagnosis of uveitis was established for 77% of patients, with the remainder classified as having idiopathic disease (Table 2). Among specific diagnoses, the most frequent were anterior herpetic uveitis (15.6%), followed by Fuchs’ uveitis (9.7%), HLA-B27-positive anterior uveitis (7.8%), and presumed tuberculous uveitis (5.7%, Fig. 1) The most frequent specific diagnoses in those aged below 18 years were Juvenile Idiopathic Arthritis (JIA) (31.9%), pars planitis (14.7%), followed by VKH and anterior herpetic uveitis (6.0%). Among adults (19–64 years), the most common specific diagnosis was anterior herpetic uveitis (14.0%), followed by Fuchs uveitis (12.3%) and HLA B27-positive anterior uveitis (9.8%). In older subjects (≥65 years), the most prevalent entities were anterior herpetic uveitis (33.3%), presumed tuberculous uveitis (14.7%), and presumed sarcoidosis (9.8%) (Fig. 2).

**Table 2:** Frequency of specific diagnoses (*n*,  %)

Specific diagnosis	*N*	%
Anterior herpetic uveitis	154	15.6
Fuchs uveitis	96	9.7
HLA-B27+anterior uveitis	76	7.7
Presumed ocular tuberculosis	56	5.7
Behçet	48	4.8
JIA	47	4.7
Toxoplasmosis	47	4.7
Presumed sarcoidosis	43	4.3
Vogt–Koyanagi–Harada	41	4.1
PICCP	40	4.0
Pars planitis	33	3.3
Anterior Cytomegalovirus uveitis	17	1.7
ARN	9	0.9
Multiple sclerosis	9	0.9
Birdshot retinochoroiditis	8	0.8
Syphilis	8	0.8
Serpiginous tuberculosis	5	0.5
Sympathetic ophthalmia	5	0.5
Tinu	5	0.5
Candida	3	0.3
Others	10	0.2

Women were considerably more likely to have had a specific diagnosis (*p* = 0.004), except for Behçet disease (56% males vs 44% females), CMV (71% males vs 29% females), and syphilis (75% males vs 35% females).

Infectious uveitis was diagnosed in 301 subjects (30% of total cases), most due to HSV (51%), followed by tuberculosis (19%), and toxoplasmosis (16%) (Table 1). Infectious uveitis was more frequent in males (34%) than females (28%) and in subjects over age 65 years (54%), compared to 12% in children and 28% in middle-age subjects (*p* < 0.001). In 150 (50%) patients with presumed infectious uveitis, ocular fluid biopsy was performed in order to confirm the clinical suspicion. Forty-seven samples (31.3%) tested positive for the following infectious agents: 18 CMV, 12 HSV, 11 Toxoplasma, 5 VZV, and 1 Listeria. Sixteen patients positive for CMV and 9 positive for HSV-1 presented with hypertensive anterior granulomatous uveitis.

In a quarter of cases, uveitis was associated with systemic diseases (259/990), with levels higher among females (29%) than males (22%, *p* = 0.016) and in the younger age subjects (44%) than those aged above 18 years (25%, *p* < 0.0001). According to anatomical classification, in those with systemic disease, panuveitis was the most prevalent (49%), followed by anterior (21%), intermediate (19%), and posterior uveitis (15%, *p* < 0.0001). The most common specific diagnoses in patients with systemic diseases were JIA and Behçet’s (*n* = 47, 18.5%), followed by presumed sarcoidosis (*n* = 43, 17%), VKH, and HLA-B27-positive anterior uveitis (*n* = 41, 16.1%).

**Fig. 3:**
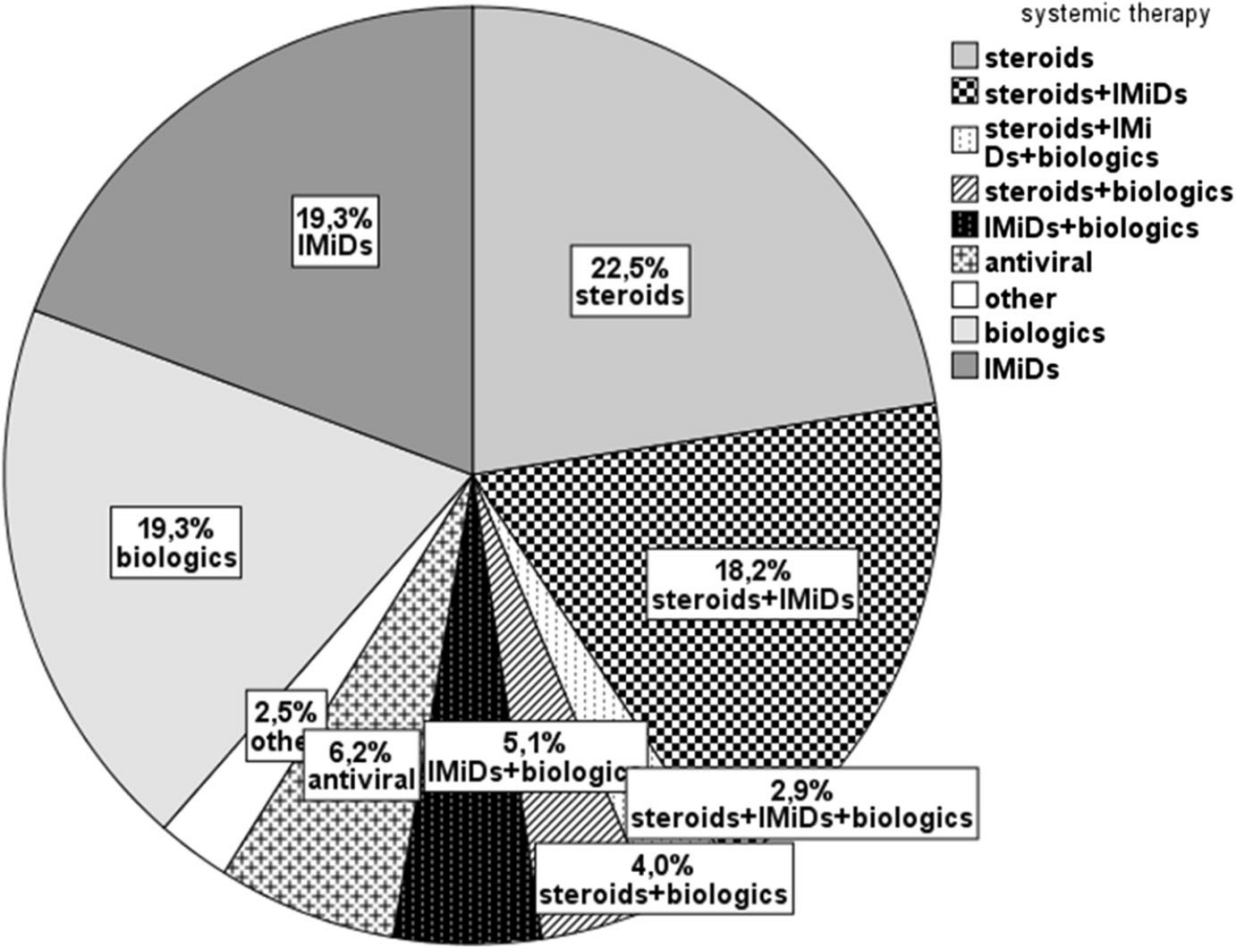
Ongoing systemic therapy (*n* = 277 cases)

With regard to treatment, 277 (28%) patients were on systemic therapy, with steroids alone (22.5%), or steroids and either immunomodulatory agents (IMiDs) (18.2%) or biologics (4.0%), or with all three of these drug groups (2.9%). Fifty-three patients (19.3%) had been treated with biologics and immunomodulatory agents alone (Fig. 3). Overall, 549 (56%) patients developed at least one complication. Cataract was the most common, occurring in 19% of cases, followed by posterior synechiae (14%), macular edema (13%), and glaucoma (11%).

## Discussion

This is only the fourth epidemiological study on uveitis in Italy [5, 21, 22], with the most recent one covering the period 2002–2008 [5]. In the same territory (533,000 inhabitants) during 24 months (2013–2015), we observed 164 new cases of uveitis, giving an estimated incidence of 15.4 cases per year per 100,000 inhabitants, higher than the value in our previous analysis in 2008 (14.6/year/100,000 people).

The distribution of cases showed the predominance of anterior uveitis, consistent with the previous studies [23–57], including our own [5]. In the last series from our site, age at presentation was significantly higher with a significant female prevalence (see Table 1). As compared to the previous series, we observed a similar frequency of anterior and intermediate uveitis (54 and 51%, and 7 and 6%, respectively) and a significant prevalence of panuveitis (in the latter study) respect to posterior uveitis (23 vs 20%, *p* < 0.001).

In this study, specific diagnoses were made in 77% of patients, higher than in other reports, [5, 23–57] which range from 42 to 75%. The increase of specific diagnoses is likely the result of both an interdisciplinary approach and the introduction of new diagnostic techniques. PCR and Antibody Index tests on eye fluids helped to confirm the diagnosis of different herpetic etiologies.

Also, the introduction of interferon gamma tests like Quantiferon helped to confirm the diagnosis of tuberculosis.

The prevalence of infectious uveitis (30%) was significantly higher (*p* = 0.002, Table 1) than in our previous series (24%): anterior herpetic uveitis, the most common specific diagnosis, increased from 9.1% in 2008 to 15.6%, while no difference was detected between the proportion with tuberculosis (4.4 in 2008 to 5.7% in current series, *p* = 0.19). Eye fluids analysis was able to confirm some specific infectious etiologies, e.g., HSV and CMV, while other diagnoses, such as Fuchs uveitis, decreased from 22.7 to 9.7% in the latter series. We observed also a lower prevalence of toxoplasmosis, from levels of 6.9% in the first analysis to 4.7% in the present analysis (Table 3).

**Table 3:** Frequencies (percentages of total cases) of key uveitis diagnoses in comparison with previous studies

Country	Author	*N*; Period	Fuchs	HLA-B27	HSV	Behçet’s	Toxoplasmosis	Sarcoidosis	TB	VKH
Italy	Present data	990; 2013–2015	9.7	7.7	15.6	4.8	4.7	4.3	5.7	4.1
	Cimino^9^	1064; 2002–2008	22.7	5.3	9.9	5.3	6.9	2.2	4.4	1.9
	Mercanti^24^	655; 1986–1993	2.1	na	11.7	3.0	17.7	0.8	7.0	1.4
	Pivetti-Pezzi^25^	1417; 1986–1993	8.3	3.4	5.3	1.3	6.6	0.2	na	2.0
UK	Jones^26^	3000; 1991–2013	11.5	4.5	1.9	2.7	6.9	9.7	3.3	0.8
Austria	Barisani-Asenbauer^27^	2619; 1995–2009	4.6	19.5	7.1	1.9	6.6	3.2	0.8	0.4
Spain	Llorenç^28^	1022; 2009–2012	1.0	13.0	12.0	5.0	7.0	3.0	5.0	1.0
Germany	Grajewski^29^	476; 2012–2013	7.0	19.0	12.0	2.0	7.1	11.0	na	0.6
	Jacob^30^	1916; 2001–2006	6.9	7.1	6.1	1.8	4.2	4.5	1.1	0
Swiss	Tran^31^	558; 1990–1993	5.4	15.9	13.8	1.1	9.5	5.9	9.5	0.2
France	Bodaghi^32^	927; 1991–1996	2.5	4.7	8.6	6.1	11.9	6.4	4.1	2.0
Japan	Nakahara^39^	695; 2010–2012	2.1	3.0	6.0	5.6	0	9.4	1.5	7.9
Saudi Arabia	Al Dhahri^18^	642; 1998–2013	11.3	9.0	10.0	8.4	6.9	4.5	17.8	19.6
Tunisia	Khairallah^47^	472; 1992–2003	3.0	2.8	11.9	12.3	10.1	1.7	1.1	4.4
Turkey	Kazokoglu^49^	761; 2004	5.1	2.4	2.8	32.1	4.7	0.9	0.3	1.1
US	Rodriguez^50^	1237; 1982–1992	2.6	3.8	7.2	2.5	4.8	9.6	0.6	0.9

Tuberculosis and syphilis accounted for 20% and almost 3% of infectious etiologies, respectively, an increase in the recent years, possibly due to the rise in immigration in Italy. The percentage of non-Italian ethnicity among patients rose from 3% in the first series to 10.7% in this report (*p* < 0.0001, Table 1).

The relatively high percentage of uveitis associated with systemic diseases (JIA, sarcoidosis, VKH, HLA-B27) is consistent with what Barisani-Asenbauer et al. reported [24], underlining the need for an interdisciplinary approach to reduce the diagnostic delay, and thus ocular as well as systemic complications due to non-appropriate therapies. In our series, 44 patients presented acute anterior non-granulomatous uveitis (38/42 HLA-B27-positive anterior uveitis) associated with ankylosing spondylitis. In 35/42 of cases, the ocular diagnosis preceded the systemic one.

The leading complications observed in our study were cataract, glaucoma, and macular edema, in line with several previous reports [8]. Regarding medications, traditional immunomodulatory agents still seem to be preferred by ophthalmologists in autoimmune conditions, rather than biologics, except for HLA-B27-associated uveitis, where anti-TNFs are prescribed more frequently.

Diagnostic delays are often due to difficulties in establishing the primary diagnosis of uveitis. Comparisons among surveys conducted in uveitis clinics elsewhere in the world often present great differences in the prevalence of diagnostic categories (Table 3). Such differences are probably related to genetic, geographical, social, and environmental factors. They may also be attributable to heterogeneity in the diagnostic criteria and definitions, together with the availability of different investigation techniques, as well as different interpretations of the anatomical categorization of intraocular inflammation.

In conclusion, the pattern of uveitis appears to be changing, very likely due to the spreading of infectious diseases, to the availability of new diagnostic tests, such as aqueous analysis by PCR and AIC, and to more refined classification (such as the PICCP grouped together on the basis of similar etiology and particular clinical and imaging aspects). Moreover, an interdisciplinary approach that includes ophthalmologists, rheumatologists, pediatricians, and infectious disease specialists with uveitis experience may help to raise the standards of treatment, and improve prognosis and clinical course of the disease, thereby reducing the risk of complications.
